# Post-translational modifications in hepatocellular carcinoma: unlocking new frontiers in immunotherapy

**DOI:** 10.3389/fimmu.2025.1554372

**Published:** 2025-02-18

**Authors:** Yuexian Piao, Naicui Zhai, Xiaoling Zhang, Wenjie Zhao, Min Li

**Affiliations:** ^1^ Department of Interventional Therapy, First Hospital of Jilin University, Changchun, China; ^2^ Core Facility of First Hospital of Jilin University, Changchun, China; ^3^ Key Laboratory of Organ Regeneration and Transplantation of Ministry of Education, First Hospital of Jilin University, Changchun, China; ^4^ National-Local Joint Engineering Laboratory of Animal Models for Human Disease, First Hospital of Jilin University, Changchun, China

**Keywords:** post-translation modification, immunotherapy, hepatocellular carcinoma, lactylation, glycosylation

## Abstract

Liver cancer, particularly hepatocellular carcinoma (HCC), is one of the most common and aggressive malignancies worldwide. Immunotherapy has shown promising results in treating HCC, but its efficacy is often limited by complex mechanisms of immune evasion. Post-translational modifications (PTMs) of proteins play a critical role in regulating the immune responses within the tumor microenvironment (TME). These modifications influence protein function, stability, and interactions, which either promote or inhibit immune cell activity in cancer. In this mini-review, we explore the diverse PTMs that impact immune evasion in liver cancer, including glycosylation, phosphorylation, acetylation, and ubiquitination. We focus on how these PTMs regulate key immune checkpoint molecules such as PD-L1, CTLA-4, and the TCR complex. Furthermore, we discuss the potential of targeting PTMs in combination with existing immunotherapies to enhance the effectiveness of treatment in HCC. Understanding the role of PTMs in immune regulation may lead to the development of novel therapeutic strategies to overcome resistance to immunotherapy in liver cancer.

## 1 Introduction

Liver cancer, particularly hepatocellular carcinoma (HCC), remains one of the most lethal malignancies worldwide, accounting for a significant proportion of cancer-related deaths (1). Despite advances in treatment modalities such as surgical resection, liver transplantation, and systemic therapies, the prognosis for advanced HCC remains dismal. The limited therapeutic options and poor survival outcomes in patients with advanced stages of the disease underscore the urgent need for more effective treatment strategies (2). Immunotherapy has emerged as a promising approach to treating various cancers, including HCC (3). Immune checkpoint inhibitors (ICIs), particularly those targeting the PD-1/PD-L1 pathway, have shown significant promise in a subset of HCC patients, offering durable responses and improved survival (4). However, the clinical success of immunotherapy in HCC has been hindered by several challenges, most notably immune evasion mechanisms within the tumor microenvironment (TME) (5). Tumor cells employ a variety of strategies to escape immune surveillance, including the upregulation of immune checkpoint proteins, secretion of immunosuppressive cytokines, and alterations in immune cell trafficking. These mechanisms collectively create a barrier that impedes the full activation and effectiveness of immune responses, limiting the potential of immunotherapy in this disease.

While much attention has been given to the role of immune checkpoints and cytokines in immune evasion, recent studies have revealed that post-translational modifications (PTMs) play a crucial and often overlooked role in regulating these immune processes (6, 7). PTMs are enzymatic modifications that occur on proteins after their synthesis, profoundly influencing their activity, stability, localization, and interactions. These modifications alter the function of immune checkpoint proteins, immune cell signaling, and immune responses, offering potential avenues to enhance the efficacy of immunotherapies. PTMs such as phosphorylation, glycosylation, acetylation, and ubiquitination regulate the function of immune checkpoint molecules like PD-1, PD-L1, and CTLA-4, which are central players in the immune evasion strategies employed by tumors (8, 9). Moreover, PTMs also affect the behavior of immune cells, such as T cells, macrophages, and dendritic cells, modulating their ability to recognize and attack tumor cells (10). As understanding of how PTMs influence immune responses in HCC increases, targeting PTM could open new therapeutic opportunities to improve immunotherapy outcomes.

This review aims to explore the emerging role of PTMs in liver cancer, specifically in immunotherapy. By examining how PTMs regulate immune checkpoint molecules, immune cell functions, and the tumor microenvironment, we will highlight the potential for PTM-targeted therapies to enhance the effectiveness of existing immunotherapies in HCC. Ultimately, this approach may pave the way for more personalized and efficient treatment strategies, offering hope for patients with this challenging and aggressive cancer.

## 2 Key post-translational modifications regulating immune responses

In cancer immunotherapy, PTMs play a crucial role in regulating immune cell functions and modulating immune escape mechanisms. Below are several PTMs most relevant to immunotherapy in HCC, highlighting their impact on immune evasion, immune checkpoint regulation, and immune cell activation.

### 2.1 Phosphorylation in liver cancer immunotherapy

Phosphorylation is one of the most prevalent PTMs and plays a critical role in the regulation of immune cell activation, proliferation, and cytokine secretion. In HCC, phosphorylation of both immune checkpoint proteins and intracellular signaling molecules is integral to immune evasion and the overall immune response to the tumor. Aberrant phosphorylation in the TME significantly impacts the efficacy of immunotherapy, especially immune checkpoint blockade (ICB).

Phosphorylation of key genes in the immune system is crucial for regulating immune response, tumor growth, and treatment resistance in liver cancer. Extracellular signal-regulated kinase (ERK) phosphorylates PD-1 at Thr234, facilitating its interaction with USP5. Conditional knockout of Usp5 in T cells enhances effector cytokine production and slows tumor growth in mice (11). Inhibiting USP5, along with Trametinib or anti-CTLA-4 treatment, shows an additive effect in inhibiting tumor growth in mice (11). Tumor-infiltrating lymphocytes (TILs) showed high levels of phosphorylated PD-1. Phosphorylation of PD-1 helps identify T cells that are not working properly (dysfunctional). Blocking PD-1 rescues these dysfunctional T cells, allowing them to become more effective at fighting cancer (12). The upregulation of PCSK9, driven by AKT-S473 phosphorylation, promotes HCC progression and contributes to sorafenib resistance. The palmitoylation of PCSK9 at cysteine 600 enhances its binding to Phosphatase and Tensin Homolog (PTEN), leading to PTEN degradation via the lysosome. This degradation results in increased AKT activation, which facilitates tumor survival and progression, ultimately affecting tumor immunotherapy (13). Moreover, the phosphorylation of STAT3, a transcription factor, is commonly observed in the TME of liver cancer and contributes to immune suppression. STX6 promotes HCC progression by activating the JAK-STAT signaling pathway. It interacts with RACK1 to recruit STAT3, which leads to the activation of STAT3 transcriptional activity. This process enhances STAT3 phosphorylation, driving tumorigenesis and immune evasion in HCC (14). Correspondingly, suppression of STAT3 phosphorylation by receptor interacting serine/threonine kinase 4 (RIPK4) or XZH-5 suppress HCC invasion and leading to cell apoptosis (15, 16).

In addition, phosphorylation determines T cell chimeric antigen receptor (CAR)-T cells activation. ZAP70 is a kinase involved in the early signaling events following T cell receptor (TCR) engagement. By decreasing ZAP70 phosphorylation, ITPRIPL1 impedes the activation of T cells during the initial phase of the immune response. This disruption in T cell activation allows the tumor to evade immune detection, which in turn promotes cancer progression (17). Similarly, phosphorylation plays a crucial role in activating CAR-T cells following antigen engagement. When the CAR binds to an antigen on a cancer cell, it creates a narrow intermembrane space that excludes the bulky phosphatase CD45. This exclusion prevents CD45 from dephosphorylating the CAR, allowing kinases to phosphorylate the intracellular domains of the CAR. Phosphorylation of the CAR then triggers downstream signaling pathways that activate the T cell, leading to a robust immune response against the target antigen (18).

### 2.2 Glycosylation in liver cancer immunotherapy

Glycosylation is a PTM with glycolic acid added to the target protein, it impacts protein function, immune cell recognition, and tumor progression. In liver cancer, altered glycosylation patterns on immune checkpoint molecules, adhesion proteins, and receptors have been shown to influence immune cell interactions, tumor immune evasion, and the response to immunotherapy (19). Glycosylation usually has two types, N-glycosylation and O-glycosylation. N-glycosylation profiling could be a useful tool for distinguishing between HCC subtypes, aiding in early detection and the development of targeted therapies (20). Inhibition of N-glycosylation decreased M2 polarization and production of chemokine CCL22 and contributes to cancer immunotherapy (21, 22). The recurrent HCC patient after liver transplantation presented significantly increased O-glycosylation (23). The role of O-glycosylation in liver cancer has been reviewed (24).

**Figure 1:**
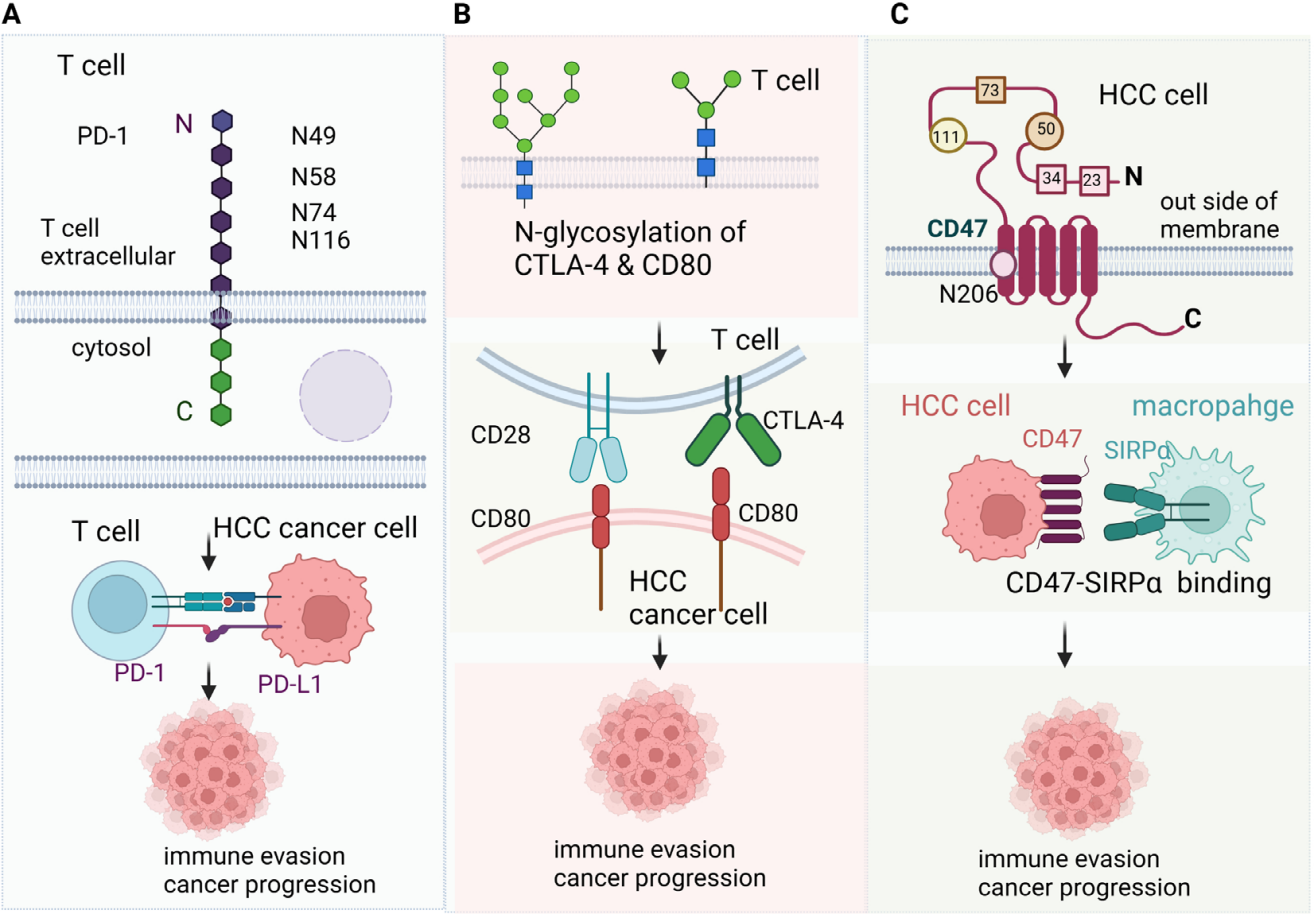
Glycosylation in liver cancer immunotherapy **(A)** PD-1 is highly N-glycosylated at sites N49, N58, N74, and N116, with N58 being particularly important. Upon T cell activation, Upon T cell activation, PD-1 glycosylation is upregulated, which enhances its interaction with PD-L1 on tumor cells, resulting in immune suppression. **(B)** CTLA-4 is glycosylated at N76, N108 and N207.The glycosylation of CTLA-4 affects its ability to bind to CD80/CD86, which in turn regulates T cell activation and the suppression of antitumor immune responses. CD28 is a key glycoprotein on T cell that binds to CD80 and CD86 to provide co-stimulatory signals for T cell activation. When N-glycosylation on CD28 is blocked or reduced, such as by mutations in the N-glycosylation site or through glycosidase inhibitors, the binding of CD28 to CD80 is significantly increased. This leads to enhanced downstream signaling, which amplifies T cell activation. **(C)** CD47 is a “don’t eat me” signal that helps tumors evade phagocytosis by macrophages (30). CD47 is overexpressed in HCC, N-glycosylation of CD47 on N23, N34, N50, N73, N111, and N206 enhance its binding to signal-regulatory protein alpha (SIRPα) on macrophages, preventing the immune system from recognizing and eliminating tumor cells.

Glycosylation of immune checkpoints are common and crucial for their function in immunotherapy (**Figure 1**). PD-1 is highly N-glycosylated at sites N49, N58, N74, and N116, with N58 being particularly important. Upon T cell activation, Upon T cell activation, PD-1 glycosylation is upregulated, which enhances its interaction with PD-L1 on tumor cells, resulting in immune suppression (25). CTLA-4 is glycosylated at N76, N108 and N207.The glycosylation of CTLA-4 affects its ability to bind to CD80/CD86, which in turn regulates T cell activation and the suppression of antitumor immune responses (26–28). CD28 is a key glycoprotein on T cell that binds to CD80 and CD86 to provide co-stimulatory signals for T cell activation. When N-glycosylation on CD28 is blocked or reduced, such as by mutations in the N-glycosylation site or through glycosidase inhibitors, the binding of CD28 to CD80 is significantly increased. This leads to enhanced downstream signaling, which amplifies T cell activation (29). CD47 is a “don’t eat me” signal that helps tumors evade phagocytosis by macrophages (30). CD47 is overexpressed in HCC, N-glycosylation of CD47 on N23, N34, N50, N73, N111, and N206 enhance its binding to signal-regulatory protein alpha (SIRPα) on macrophages, preventing the immune system from recognizing and eliminating tumor cells (31, 32). 2B4 (CD244) is a glycoprotein expressed on NK cells, CD8^+^ T cells, and dendritic cells, playing a crucial role in regulating their function through interactions with CD48. N-glycosylation and sialylation of 2B4 are essential for its binding to CD48, and inhibiting glycosylation or removing sialic acid enhances 2B4/CD48 binding, leading to increased NK cell activation (33). In addition, N-glycosylation of Mer tyrosine kinase (MerTK) stabilize MerTK and promotes HCC progression (34). Glypican-3 is a member of the glypican family and has been demonstrated a therapeutic target of HCC, the glycosylation of Glypican-3 stabilizes it and promotes HCC progression (35).

### 2.3 Acetylation in liver cancer immunotherapy

Acetylation is a PTM where an acetyl group (COCH3) is added to a protein, typically at lysine residues, altering its function, stability, or interaction with other molecules. By affecting transcriptional activity and immune cell metabolism, acetylation regulates immune cell activation, T-cell tolerance, and overall antitumor immunity. Acetylation plays a key role in regulating immune response and tumor progression, where curcumin inhibits histone acetylation by reducing p300-induced acetylation at the TGF-β1 promoter region, thus modulating the expression of immune-related genes and enhancing the immune response when combined with anti-PD-1 in HCC treatment (36).

The NF-κB signaling pathway is a central regulator of immune responses, inflammation, and tumor progression. Acetylation of NF-κB proteins, such as p65 (RelA), activates the pathway, leading to the upregulation of pro-inflammatory cytokines, chemokines, and immune effector molecules (37). This inflammatory milieu promotes tumor cell survival, immune evasion, and resistance to therapies. However, targeting NF-κB acetylation in liver cancer could help to reverse immune suppression and improve the effectiveness of immunotherapies by restoring immune surveillance. Acetylation of NF-κB subunit p65 at site Lys310(p65^Lys310^) by p300 in macrophages mediates anti-inflammatory property by Berberine (38). TNF receptor-associated factor 6 (TRAF6) is an adaptor protein that is involved in the activation of NF-κB and MAPK signaling pathways, which regulate immune responses. In HCC, TRAF6 ubiquitination promotes the activation of these pathways, leading to chronic inflammation and immune suppression within the TME. TRAF6 promotes HCC by interacting with HDAC3 to enhance c-Myc expression and protein stability (39).

Acetylation also regulates the function of immune checkpoint proteins like PD-1 and CTLA-4, which are critical in mediating immune tolerance and suppression. In HCC, acetylation of these checkpoint proteins alters their stability and activity, contributing to immune evasion. For example, acetylation of PD-L1 by p300 enhances its nuclear translocation, where it regulates immune-response-related gene expression, and inhibiting this acetylation process blocks PD-L1’s nuclear translocation, reprograms gene expression, and boosts the effectiveness of PD-1/PD-L1 blockade in cancer immunotherapy (40).

### 2.4 SUMOylation in liver cancer immunotherapy

SUMOylation, the addition of small ubiquitin-like modifiers (SUMOs) to proteins, is a key PTM that regulates protein stability, localization, and activity. In HCC, SUMOylation plays an important role in modulating immune responses by altering protein-protein interactions and influencing the transcriptional activity of immune-related factors.

In HCC, SUMOylation of NF-κB subunits, such as p65 (RelA), suppress their transcriptional activity and suppress tumor progression (41). Small ubiquitin-related modifier 1 (SUMO1) promotes the translocation of mesencephalic astrocyte-derived neurotrophic factor (MANF) into the cell nucleus and increases the interaction between MANF and p65, thus inhibit NK-kB transcription and inflammatory response, mutation of p65 motifs for SUMOylation abolished the interaction of p65 and MANF (42). Interleukin-33 (IL-33) functions both as a secreted cytokine and as a nuclear factor, with pleiotropic roles in cancer and immunity. SUMOylated IL-33 in the nucleus stabilizes IRF1 in HCC to promote cancer cell immune escape (43). Moreover, the tumor suppressor protein p53 is involved in regulating cellular stress responses and immune surveillance. In HCC, SUMOylation of p53 has been shown to affect its stability and function. p53-stabilizing and activating RNA (PSTAR) enhances the SUMOylation of heterogeneous nuclear ribonucleoprotein K (hnRNP K), which strengthens the interaction between hnRNP K and p53. This interaction is crucial for the stabilization and activation of p53, which leads to cell cycle arrest and suppression of tumorigenicity in HCC cells (44). Export-5 was highly expressed in HCC and it was SUMOylated by SUMO2 at K125 to promote the HCC progression (45). RING-type E3 ubiquitin ligase RNF146 is a key regulator of the Wnt/β-catenin signaling pathway, which is frequently hyperactivated in HCC SUMOylation of RNF146 leads to activation of Wnt/β-Catenin pathway to promote HCC progression (46). The SUMO activating enzyme SAE1 promotes HCC by enhancing mTOR SUMOylation (47). In addition, SUMOylation has a crosstalk with phosphorylation in HCC progression and immune evasion (48).

### 2.5 Lactylation in liver cancer immunotherapy

Lactylation, a modification with the addition of lactate to lysine residues of proteins, driven by the elevated lactate levels in TME has emerged as a key player in metabolic adaptation and immune regulation in HCC (49). A lactylome analysis has revealed that lactylation is critical for metabolic adaptation in HCC, where lactate accumulation in TME leads to the modification of various proteins involved in cellular metabolism (50). These metabolic shifts support tumor growth and survival by enhancing the Warburg effect and promoting immune evasion. Moreover, lactylation has been shown to regulate gene expression, with lactylation-related gene signatures being capable of predicting prognosis and treatment responsiveness in HCC patients (51). The role of lactylation extends beyond metabolic regulation to influencing immune cells in the TME. Hepatic stellate cells, a key component in liver fibrosis and cancer progression, have been shown to promote HCC development through the regulation of histone lactylation (52). Hexokinase 2-mediated gene expression via histone lactylation is required for hepatic stellate cell activation and liver fibrosis (53). Single-cell RNA sequencing and spatial transcriptomics analyses have provided insights into how lactylation in these cells contribute to tumor progression by modulating gene expression that facilitates immune evasion and tumor growth. Additionally, lactylation has been implicated in the activation of signaling pathways that enhance the immunosuppressive environment in HCC, creating challenges for immune-based therapies.

Lactylation on immune cell function, such as regulatory T cells (Tregs), has been highlighted in recent studies. Tumor metabolites like lactate modulate lactylation in Tregs, enhancing TGF-β signaling and promoting immune tolerance (54). This modification not only supports immune evasion but also exacerbates the resistance to immunotherapies. Furthermore, therapeutic strategies targeting lactylation have begun to emerge. SIRT3, a deacetylase enzyme, mediates the delactylation of proteins like cyclin E2, thus preventing HCC growth. Inhibition of histone lactylation, such as through the action of royal jelly acid on H3K9la and H3K14la sites, has been demonstrated to suppress tumorigenicity (55, 56). Additionally, global profiling of protein lactylation in HCC has identified specific proteins that are modified by lactylation, offering new targets for intervention and enhancing the precision of treatment approaches (57).

Lactylation also regulate other important transcriptional factors and cytokines related to immunotherapy (58). NF-κB is an important pro-inflammatory signaling pathway in HCC, and lactylation regulates the expression of inflammation-related genes by modifying specific histone sites. Studies have shown that increased lactate metabolism induces lactylation of histone H3K18, thereby activating NF-κB target genes such as IL-6 and TNF-α (59). Lactylation enhances the binding ability of the NF-κB p65 subunit and increases its recruitment to the promoters of pro-inflammatory genes. The high lactate and lactylation level inhibit the efficacy of Lenvatinib trough regulating PD-L1 expression on neutrophil in HCC (59).This regulation may promote tumor cell proliferation and suppress immune cell activity.

**Figure 2:**
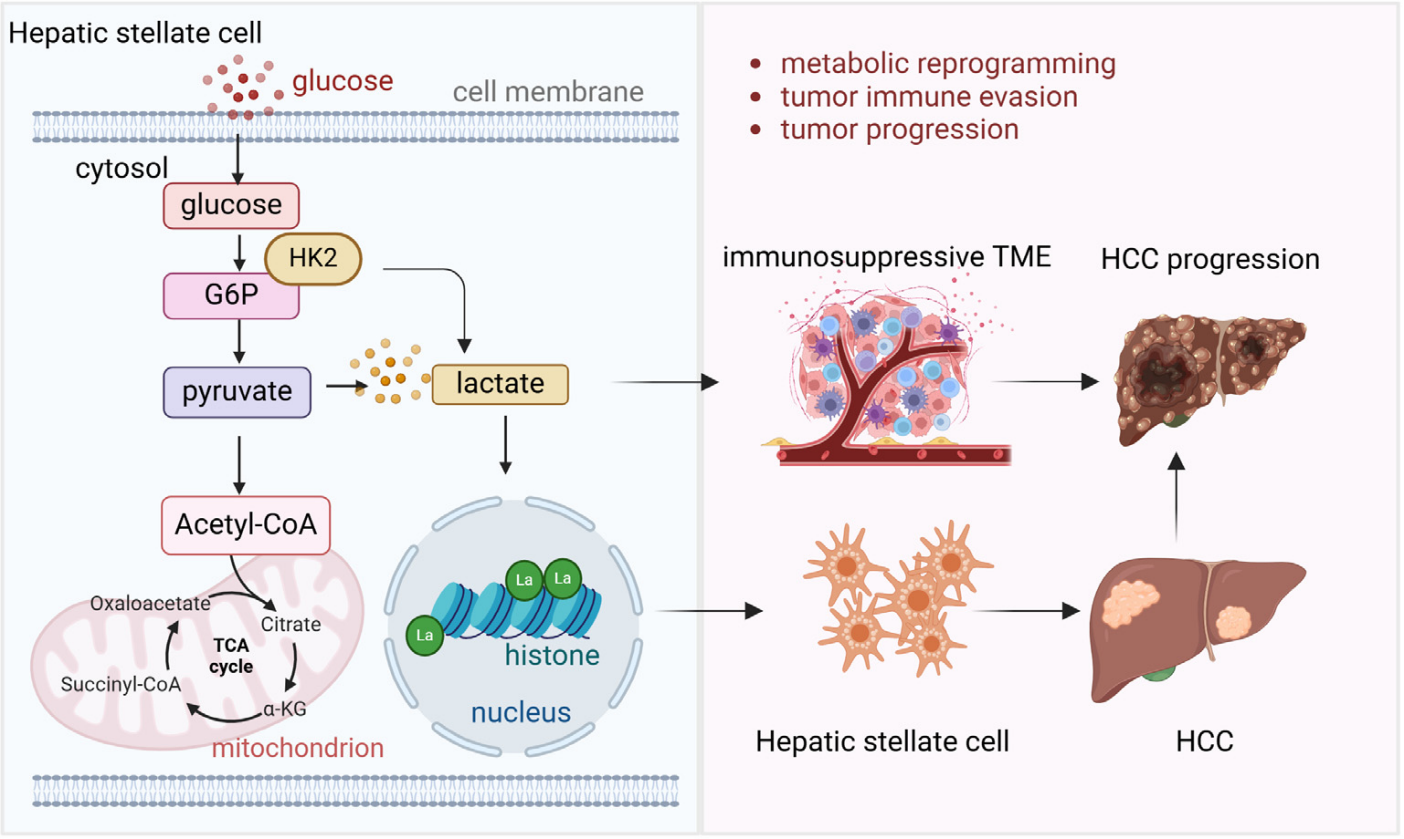
Lactylation and liver cancer progression and immunotherapy Hepatic stellate cell requires histone lactylation mediated by Hexokinase 2 (HK2) to activate and develop into fibrosis and further progress into HCC. High energy request of HCC requires glycolysis and more lactate is produced in this process. High lactate level leads to the immunosuppressive tumor microenvironment which facilitates tumor cell immune evasion and HCC progression.

Phosphorylation, glycosylation, SUMOylation, and lactylation are PTMs that play crucial roles in HCC. Phosphorylation regulates the activity of glycosyltransferases, influencing the glycosylation of key immune checkpoint molecules such as PD-L1, which enhances its stability and immunosuppressive functions. Conversely, glycosylation modulates phosphorylation cascades by altering receptor conformations, thereby amplifying or suppressing downstream signaling. Phosphorylation and SUMOylation often act synergistically through phosphorylation-dependent SUMOylation sites, as seen in transcription factors that require prior phosphorylation for SUMO conjugation, further impacting immune signaling pathways like NF-κB. Lactylation, derived from lactate metabolism, is closely linked to glycosylation, as both are influenced by the metabolic state of tumor cells. Lactylation regulates the expression of enzymes involved in glycosylation, while elevated lactate levels enhance the stability of glycosylated proteins in TME. Furthermore, lactylation and SUMOylation jointly modulate transcriptional programs that govern immune suppression, such as through STAT3 activity. Together, these PTMs form a complex regulatory network that drives immune evasion and promotes HCC progression. Understanding their interplay offers valuable insights into potential therapeutic interventions targeting multiple PTM pathways (**Figure 2**).

## 3 Targeting PTMs to enhance immunotherapy in HCC

### 3.1 Modification of PTM of immune checkpoints

In HCC, PD-L1 is often overexpressed and undergoes glycosylation, which enhances its stability and interaction with the immune system. Inhibiting PD-L1 glycosylation enhances the efficacy of PD-1/PD-L1 inhibitors, such as nivolumab and pembrolizumab, by improving T-cell activation and promoting immune surveillance in the TME (60). This strategy could potentially lead to better clinical outcomes for HCC patients who are resistant to current immunotherapies. In HCC, the phosphorylation of CTLA-4 plays a pivotal role in regulating T cell function and immune tolerance (61). Modulating CTLA-4 phosphorylation could restore T cell activity and enhance the immune response against tumor cells. Developing small molecules or biologics that target specific kinases or phosphatases responsible for regulating CTLA-4 phosphorylation could improve the effectiveness of CTLA-4-based therapies. Meanwhile, targeting the PTMs of key signaling molecules involved in TCR activation, such as ZAP70 and LAT, could restore T cell activity, enhance tumor recognition, and improve the anti-tumor immune response (62).

### 3.2 Combination therapies

Combining PTM-targeting agents with traditional immunotherapies, such as immune checkpoint inhibitors or adoptive T-cell therapies, may offer synergistic effects. At present, the best available first-line treatment for advanced HCC is a combination of PDL1 blockade with atezolizumab and VEGF blockade with bevacizumab (63).Combining PD-L1 glycosylation inhibitors with PD-1/PD-L1 blockade could further enhance T-cell activation and immune response (64). Similarly, combining CTLA-4 phosphorylation modulators with CTLA-4-based therapies could restore T cell function, amplifying the overall anti-tumor immune response (65, 66). Additionally, combining PTM-targeting agents with adoptive T-cell therapies could improve T-cell persistence and efficacy (67, 68). These combination strategies hold great promise for improving clinical outcomes in HCC by overcoming immune evasion and enhancing the body’s natural immune response to tumors.

Targeting PTMs in HCC faces several translational hurdles. One major challenge is the complexity and heterogeneity of PTMs, which vary significantly across tumors and patients, complicating the development of universally effective therapies (69, 70). This variability makes it difficult to predict how PTM-targeting treatments will perform in clinical settings. Furthermore, the lack of reliable biomarkers to monitor PTM alterations during treatment hampers the ability to assess therapeutic efficacy and guide clinical decisions. Another hurdle is overcoming the resistance mechanisms driven by PTM modulation, such as immune evasion through PTM-driven regulation of immune checkpoint molecules like PD-L1 and CTLA-4. To address these challenges, strategies could include the identification of specific PTMs that drive tumor progression through advanced proteomic and genomic profiling, enabling personalized treatments (71). Combination therapies that target PTMs alongside immune checkpoint inhibitors or targeted therapies could enhance the immune response and prevent resistance (72). Additionally, improving methods for real-time monitoring of PTM changes in tumors, such as through liquid biopsy or advanced imaging techniques, could provide dynamic insights into treatment responses and resistance. By combining PTM-targeting approaches with innovative drug delivery systems, such as nanoparticles or antibody-drug conjugates, it may be possible to increase therapeutic efficacy and overcome the barriers to translation in HCC treatment.

Regarding to clinical trials, there are no PTM inhibitors in clinical trial of HCC right now. However, PTM inhibitors has been studied a lot in other tumor models. Bortezomib is a proteasome inhibitor applied in phase III clinical trial in leukemia and lymphoma (73). A novel proteasome deubiquitinase inhibitor, VLX1570 has been applied in a phase I clinical tiral in refractory multiple myeloma (74). Ruxolitinib, a JAK1/2 inhibitor, has been applied in phase III clinical trial for native patients with myelofibrosis (75).

## 4 Conclusion and future perspectives

PTMs play a pivotal role in regulating immune responses within HCC and influence the behavior of key proteins involved in immune evasion and tumor progression. These modifications impact the expression and function of critical immune checkpoint molecules, the infiltration of immune cells into TME, and overall immune system functionality. As a result, targeting PTMs represents a novel and promising strategy to enhance the efficacy of existing immunotherapies in liver cancer. The current research indicates that PTMs significantly modulate the immune landscape in HCC. By manipulating these modifications, it is possible to restore immune cell activity, improve immune checkpoint blockade effectiveness, and reduce tumor immune evasion. Strategies aimed at targeting specific PTMs could not only enhance the function of immune checkpoint inhibitors but also offer novel combinatory therapies to improve clinical outcomes in HCC patients.

Despite the promising potential of targeting PTMs, challenges remain in translating these findings into effective clinical therapies. Identifying the precise PTMs involved in immune regulation in HCC, understanding their context-dependent roles, and developing specific agents to modulate these modifications are essential steps in moving forward. Furthermore, overcoming the complexity of the TME, which includes immune suppression and metabolic dysfunction, will be crucial in improving the effectiveness of PTM-targeting therapies. Below questions dive deeper into the role of PTMs in regulating immune responses and their potential therapeutic implications in HCC immunotherapy.

1. How do different types of post-translational modifications individually or synergistically regulate immune evasion mechanisms in HCC?
2. What specific PTMs of immune checkpoint molecules are linked to resistance to immunotherapy in HCC, and how can these modifications be targeted therapeutically?
3. How can the modulation of PTMs in HCC tumor antigens enhance T cell activation or reduce tumor immune evasion?
4. What are the underlying mechanisms that regulate PTMs in the HCC tumor microenvironment, and how do these mechanisms affect the success of immunotherapy?

With advancements in precision medicine, including the use of patient-specific biomarkers and PTM-targeting agents, there is hope for significantly improving the prognosis of liver cancer patients. Personalized treatment approaches that incorporate PTM modulation could lead to better outcomes, addressing the limitations of current therapeutic options. Ultimately, continued research and clinical trials will be necessary to fully understand the role of PTMs in HCC and harness their potential to revolutionize the landscape of liver cancer immunotherapy.
